# Mid-Term Results of Ceramic Monoblock Acetabular Cups in Primary Total Hip Arthroplasty: A Minimum 5-Year Follow-Up

**DOI:** 10.3390/jcm15041672

**Published:** 2026-02-23

**Authors:** Chan Young Lee, Gong-Yeong Kim, Taek-Rim Yoon, Kyung-Soon Park

**Affiliations:** 1 Department of Orthopedic Surgery, Chonnam National University Hwasun Hospital, Hwasun-gun 58128, Jeollanam-do, Republic of Korea; cylee0167@naver.com (C.Y.L.);; 2 Department of Orthopedic Surgery, Chonnam National University Hospital, Gwangju 61469, Jeollanam-do, Republic of Korea; 3 Department of Orthopedic Surgery, Chonnam National University Medical School, Hwasun-gun 58128, Jeollanam-do, Republic of Korea

**Keywords:** total hip arthroplasty, ceramic-on-ceramic, monoblock cup, acetabular component, mid-term outcome

## Abstract

**Introduction:** Ceramic-on-ceramic (CoC) articulation in total hip arthroplasty (THA) offers excellent wear characteristics but carries risks such as liner malseating and ceramic fracture. To solve these problems, monoblock acetabular cups with preassembled ceramic liners were developed to minimize technical errors and allow the use of larger femoral heads. This study aimed to evaluate the mid-term clinical and radiological outcomes of a ceramic monoblock acetabular cup system. **Methods:** A retrospective analysis was performed on 106 primary THAs in South Korean patients using the Maxera monoblock cup (Zimmer Biomet) between 2015 and 2018, with a minimum follow-up of 5 years. Clinical outcomes were assessed using the Harris Hip Score (HHS), Western Ontario and McMaster Universities Osteoarthritis Index (WOMAC), and Visual Analog Scale (VAS). Radiologic evaluation included osteolysis and radiolucent lines. Normality of clinical variables was confirmed, and pre-to-postoperative comparisons were performed using paired t-tests. **Results**: The mean follow-up was 6.8 ± 1.4 years. The most common preoperative diagnosis was avascular necrosis (66.0%). Cups sized ≤52 mm were used in 80.2% of hips, allowing the frequent use of large femoral heads (32–40 mm). Clinical scores improved significantly: HHS from 37.0 ± 13.4 to 90.8 ± 6.2, WOMAC from 66.6 ± 11.5 to 7.6 ± 6.7, and VAS from 6.45 ± 1.1 to 1.1 ± 0.8 (*p* < 0.001). No osteolysis was observed. Radiolucent lines was appeared in four hips (3.7%) without evidence of migration or loosening. One cup fixation failure (0.9%) required revision. No cases of ceramic fracture, squeaking, or dislocation occurred. **Conclusions**: The ceramic monoblock acetabular cup demonstrated excellent mid-term clinical and radiological outcomes with a very low complication rate. The ability to reliably use large femoral heads likely contributed to enhanced joint stability. However, the absence of screw fixation and inability to directly visualize cup insertion require careful attention during cup impaction. Long-term studies with comparative cohorts are warranted.

## 1. Introduction

Total hip arthroplasty (THA) remains one of the most successful orthopedic procedures worldwide, with continuously increasing procedural volumes driven by aging populations and expanded surgical indications. In parallel with this growth, fixation strategies have evolved considerably over the past two decades. Contemporary registry-based studies have demonstrated a clear shift toward uncemented fixation in primary THA. For example, a nationwide registry-based analysis reported that uncemented fixation accounted for 56.8% of primary hip arthroplasties between 2001 and 2024, with a continuous increase in the uncemented fixation gradient over time, rising from 0.32 to 3.43. This shift was observed across all age groups, including elderly patients, and was accompanied by a stable or slightly decreased revision burden. These findings reflect a global trend toward biologic fixation and underscore the ongoing evolution of acetabular component design and implantation techniques [1]. Within this evolving landscape of fixation strategies, ceramic bearing surfaces—particularly ceramic-on-ceramic articulations—have been increasingly adopted to enhance wear resistance and long-term implant durability.

Ceramic-on-ceramic (CoC) articulation has been widely adopted in total hip arthroplasty (THA) due to its exceptional wear resistance, chemical inertness, and low osteolytic potential, making it particularly suitable for younger and active patients who require durable long-term implant performance [2]. Since its introduction in the 1970s, progressive improvements in ceramic purity, crystalline orientation, and composite reinforcement—most notably the development of BIOLOX Delta—have significantly increased fracture toughness and reduced the risk of catastrophic failure. Multiple long-term studies and national joint registries have reported excellent survivorship with modern ceramic bearings, with fracture rates now generally under 0.001–0.021%, demonstrating marked improvement compared with earlier generations [2,3,4,5].

Despite these advances, ceramic bearings remain sensitive to technical factors. Although rare, ceramic liner fracture continues to be a concern and is often associated with mechanical impingement, edge loading, or, most critically, liner malseating during insertion of modular acetabular systems [6,7]. Even subtle rotational malalignment or incomplete seating can generate uneven contact stresses, predisposing the liner to chipping or delayed catastrophic failure. Similarly, phenomena such as audible squeaking—though usually benign—have been linked to component malposition, lubrication disruption, or microseparation, highlighting the delicate interaction between implant design, material properties, and surgical technique [8,9,10].

To address the mechanical vulnerabilities of modular liner constructs, monoblock acetabular cups were developed. These implants incorporate a ceramic liner permanently bonded to a titanium shell during manufacturing, thereby eliminating the risk of intraoperative malseating and creating a rigid, monolithic structure [11]. From a biomechanical perspective, monoblock cups remove the modular interface, reducing the potential for micromotion, backside wear, and localized stress concentrations at the liner–shell junction. From a biomechanical perspective, monoblock configurations eliminate the modular liner–shell interface, which may reduce micromotion and stress concentration at the liner–shell junction. Furthermore, the ability to integrate larger ceramic femoral heads—even in smaller shell sizes—enhances stability by increasing jump distance and reducing impingement risk [12].

Although monoblock ceramic acetabular cups offer several theoretical advantages from both material and design perspectives, clinical evidence regarding their independent performance remains relatively limited. Previous studies have reported favorable short- to mid-term outcomes; however, most available data originate from Western populations, and evidence from Asian cohorts remains scarce [11,12]. Given the anatomical characteristics, lifestyle differences, and varying implant selection patterns observed in Asian patients, additional region-specific clinical data are necessary to better understand the performance and applicability of monoblock ceramic designs in this population.

Given these uncertainties, further evaluation of monoblock ceramic cups is warranted to clarify their clinical reliability and radiographic performance over time, particularly in Asian populations. The present study aimed to investigate the mid-term outcomes of primary THA using a ceramic monoblock acetabular cup, with a minimum follow-up of five years, focusing specifically on functional improvement, radiographic stability, and implant-related complications. By providing detailed clinical and radiologic data from a consecutive Asian cohort, this study contributes meaningful evidence to the growing understanding of monoblock ceramic acetabular systems and their performance across diverse patient populations.

## 2. Methods

### 2.1. Patients

**Figure 1:**
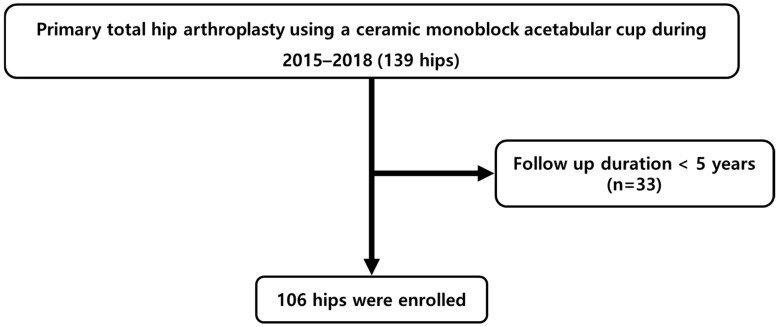
Flowchart of patient selection.

This retrospective observational study was conducted at a single tertiary medical center and included South Korean patients who underwent primary total hip arthroplasty (THA) using the Maxera monoblock acetabular cup (Zimmer Biomet, Warsaw, IN, USA) between January 2015 and December 2018. A total of 139 hips were initially screened, and 33 hips with less than 5 years of clinical follow-up were excluded, resulting in 106 hips available for final analysis (Figure 1).

### 2.2. Surgical Procedure

All operations were performed by experienced hip surgeons using either the minimally invasive two-incision (MIS-2-incision) approach or the posterolateral approach. The choice between the MIS-2-incision and posterolateral approach was primarily based on patient-specific anatomical and clinical factors rather than solely on surgeon preference. The posterolateral approach was preferentially selected in cases with significant limb length discrepancy (>2 cm), severe deformity (such as developmental dysplasia of the hip or sequelae of septic hip), body mass index (BMI) ≥ 35 kg/m^2^, or the presence of previous surgical scars around the hip. In the absence of these factors, the MIS-2-incision approach was performed according to the surgeon’s standard practice. After acetabular preparation with sequential hemispherical reamers, the Maxera monoblock cup was inserted. Because this implant does not contain screw holes and therefore cannot be attached to conventional cup holders, a dedicated edge-hook insertion handle supplied by the manufacturer was used. This device engages the circumferential rim of the metal shell and allows controlled insertion during impaction. After achieving an initial press-fit, the final impaction was performed using a monoblock-specific final impactor to optimize seating and alignment. The lack of screw holes precludes fixation augmentation and limits intraoperative visualization of insertion, necessitating meticulous tactile and radiographic assessment during implantation. Femoral stems used included ML Taper, Wagner Cone, or Fitmore stems according to canal morphology.

### 2.3. Clinical and Radiologic Evaluation

Baseline demographic variables (age, sex, body mass index, diagnosis, approach, and operated side) and implant data were collected. Clinical outcomes were assessed preoperatively and at the most recent follow-up using the Harris Hip Score (HHS), Western Ontario and McMaster Universities Osteoarthritis Index (WOMAC), and Visual Analog Scale (VAS) for pain. Radiologic evaluation was conducted using anteroposterior pelvic and cross-table lateral radiographs at each follow-up visit. Osteolysis was defined as any new or progressive radiolucency ≥2 mm. Radiolucent lines were assessed according to the DeLee–Charnley zones [13]. Postoperative complications were comprehensively reviewed, including cup fixation failure, aseptic loosening, stem complications, infection, periprosthetic fracture, squeaking, dislocation, and ceramic fracture. The medial acetabular wall–cup gap was measured on standardized anteroposterior pelvic radiographs using the *INFINITT M6 PACS* (INFINITT Healthcare Co., Seoul, Republic of Korea). The built-in digital measurement tool was used to determine the shortest perpendicular distance between the medial cortical surface of the acetabulum and the medial edge of the acetabular cup. Measurements were obtained directly from calibrated digital radiographs displayed within the PACS system. All measurements were performed independently by two orthopedic surgeons who were not involved in the index surgery. Interobserver reliability was assessed using the intraclass correlation coefficient (ICC), which demonstrated good agreement between observers (ICC = 0.87).

### 2.4. Statistical Analysis

Continuous variables were assessed for normality using the Shapiro–Wilk test. HHS, WOMAC, and VAS scores met normal distribution criteria and were compared using paired t-tests to evaluate changes between preoperative and final follow-up assessments. Categorical variables were summarized as frequencies and percentages. A *p*-value <0.05 was considered statistically significant. All statistical analyses were performed using standard statistical software (e.g., SPSS version 25.0; IBM Corp., Armonk, NY, USA).

## 3. Results

A total of 106 hips in 106 patients were included in the final analysis, with a mean age of 53.1 years (range, 18–85 years) and a mean body mass index of 24.9 ± 4.0 kg/m^2^. The average follow-up duration was 6.8 ± 1.4 years. The most common preoperative diagnosis was avascular necrosis of the femoral head, accounting for 66.0% of cases, followed by osteoarthritis (18.9%) and femoral neck fracture (7.5%). The MIS-2-incision approach was used in 90.6% of hips, reflecting the institution’s preference toward minimally invasive surgical techniques (Table 1).

Cup sizes of 52 mm or smaller were used in more than 80% of patients, yet large ceramic femoral heads (36–40 mm) were still frequently utilized due to the design characteristics of the Maxera monoblock cup. ML Taper stems were used in the majority of cases (Table 2).

**Figure 2:**
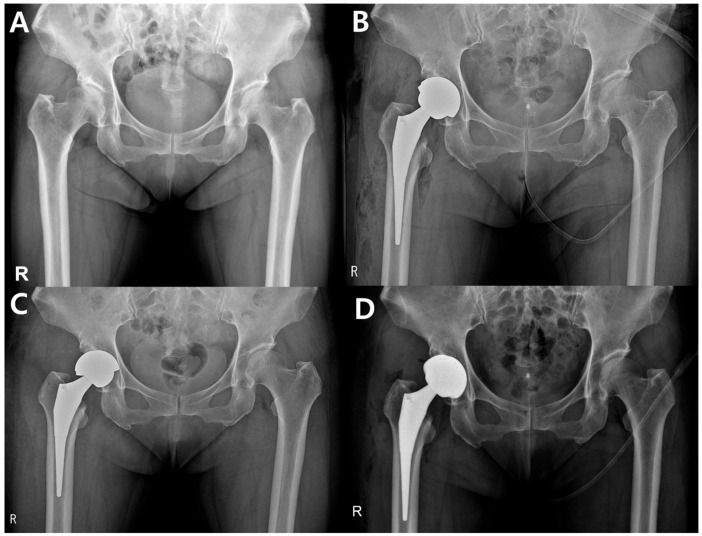
Early acetabular cup fixation failure case. (**A**) Preoperative radiograph of a 61-year-old female with right hip osteoarthritis. (**B**) Primary total hip arthroplasty using a ceramic monoblock acetabular cup (Maxera). (**C**) Early acetabular cup fixation failure observed on postoperative day 7. (**D**) Revision total hip arthroplasty using an acetabular component with a modular ceramic liner. The letter “R” in the lower left corner indicates the right side.

Clinical outcomes demonstrated significant improvements across all measures. The mean HHS increased from 37.0 ± 13.4 preoperatively to 90.8 ± 6.2 at the final follow-up (*p* < 0.001). Similarly, the WOMAC score improved from 66.6 ± 11.5 to 7.6 ± 6.7 (*p* < 0.001), and the VAS score decreased from 6.45 ± 1.1 to 1.1 ± 0.8 (*p* < 0.001), reflecting substantial pain reduction and functional recovery. Only one patient (0.9%) experienced early cup fixation failure within the first postoperative week, requiring revision surgery (Figure 2). Other complications included one case each of periprosthetic femoral fracture, femoral stem ingrowth failure, and deep infection (all 0.9%). Notably, no dislocations, ceramic fractures, or squeaking phenomena were encountered during follow-up (Table 3).

Radiographic evaluation revealed no cases of osteolysis during the entire follow-up period. Radiolucent lines were observed in four hips (3.7%), appearing in DeLee–Charnley zones 1 or 2, but these findings did not progress over time and were not associated with component loosening or migration. In addition to standard radiographic evaluation, the adequacy of cup impaction was indirectly assessed by measuring the distance between the medial surface of the acetabulum and the medial aspect of the acetabular cup on postoperative anteroposterior pelvic radiographs. As shown in Table 4, direct contact between the cup and the medial acetabular wall was observed in 92 hips (86.8%), while a measurable gap was present in 14 hips (13.2%). Among the hips which gap presents, the mean distance was 3.17 ± 1.44 mm, suggesting that satisfactory medial seating was achieved in the majority of cases. (Table 4) Subgroup analysis comparing hips with and without a medial gap revealed no significant differences in final HHS, WOMAC, or VAS scores (all *p* > 0.05). Although radiolucent lines were more frequently observed in the gap group (14.3% vs. 2.2%), this difference did not show statistical significance (*p* = 0.084) (Table 5).

## 4. Discussion

This study demonstrated excellent mid-term clinical and radiologic outcomes following THA using a ceramic monoblock acetabular cup. Significant improvements in HHS, WOMAC, and VAS scores reflect substantial pain reduction and functional recovery, consistent with previously reported benefits of ceramic-on-ceramic articulations. The absence of osteolysis in this cohort aligns with the well-documented wear resistance of modern ceramic bearings, which generate minimal particulate debris compared with polyethylene-based constructs. These findings reinforce the biological advantages of ceramic materials, particularly in younger or active patients in whom long-term implant durability is crucial.

The monoblock design may contribute to these favorable outcomes by addressing specific limitations associated with modular cup systems. Liner malseating is a known mechanism of ceramic liner fracture in modular designs, as even subtle malalignment during insertion can create asymmetric loading and predispose the liner to chipping or catastrophic fracture [14,15,16]. By eliminating the modular interface, monoblock cups inherently remove this risk, providing a more stable ceramic–metal construct. In addition, monoblock cups enable consistent use of large-diameter ceramic heads regardless of shell size, improving jump distance and thereby reducing the risk of instability. This theoretical advantage was reflected in our study, as large femoral heads (≥36 mm) were frequently used even in smaller cups without a single case of postoperative dislocation [17].

The low complication rate observed in this cohort further supports the clinical reliability of monoblock ceramic designs. Only one case of early cup fixation failure (0.9%) occurred, which may be attributable to the design’s inherent limitation of not permitting screw augmentation or direct intraoperative visualization through screw holes. Although fixation failure of monoblock acetabular cups has rarely been reported, Blakeney et al. reported a small proportion of revisions due to primary fixation failure in a series of large-diameter ceramic-on-ceramic total hip arthroplasties [18]. When compared with recent Western data on monoblock ceramic acetabular components, our findings appear broadly consistent. Jansegers et al. reported a survivorship of 96.8% at 8 years with no cases of aseptic cup loosening, while registry data from the Australian Orthopaedic Association National Joint Replacement Registry demonstrated a 12-year cumulative revision rate of 4.1% for monoblock ceramic cups, with low rates of ceramic fracture and dislocation-related revision. In our South Korean cohort with a mean follow-up of 6.8 years, only one early fixation failure (0.9%) occurred, with no cases of ceramic fracture, dislocation, or osteolysis, suggesting comparable mid-term performance in an Asian population [11,12].

However, previous studies have largely focused on bearing-related complications and implant survivorship, with limited attention to technical aspects of cup seating or impaction depth [11,12,19,20]. In the present study, the occurrence of early fixation failure in a hip with a measurable medial wall–cup gap prompted further evaluation of medial seating adequacy. Although this isolated case raises awareness of a potential technical consideration during implantation, subgroup analysis demonstrated no significant differences in clinical or radiographic outcomes between hips with and without a measurable gap. These findings suggest that the presence of a small medial gap alone does not necessarily compromise mid-term implant stability. Rather, early fixation failure is likely multifactorial and may reflect a combination of surgical technique, bone quality, and initial press-fit stability. Accordingly, meticulous acetabular preparation and careful assessment of press-fit fixation remain essential when implanting monoblock acetabular components. Given the limited ability to directly visualize seating in monoblock designs, routine intraoperative fluoroscopic assessment may provide an additional safety measure to ensure adequate cup depth and positioning during impaction. Notably, no ceramic fractures or squeaking events occurred in our series. These findings are consistent with biomechanical improvements seen in contemporary ceramics such as BIOLOX Delta, which exhibit superior fracture toughness and reduced susceptibility to edge loading.

Radiographic findings were similarly favorable, with no cases of osteolysis and only a small number of non-progressive radiolucent lines. The clinical significance of isolated radiolucent lines in ceramic constructs remains uncertain; however, the absence of progression or component migration in all cases suggests stable osseointegration and favorable host–implant interaction.

Another important aspect of this study is that it provides data from an Asian cohort, which is underrepresented in existing literature on monoblock ceramic cups. Anatomical differences, lifestyle factors, and implant selection patterns may influence outcomes, and region-specific evidence is essential to validate the generalizability of monoblock designs across diverse populations. In our cohort, the mean body mass index (BMI) was 24.9 kg/m^2^. According to Asian BMI criteria, this value corresponds to the upper range of normal or borderline overweight, which differs from Western classification standards. Therefore, the generally favorable outcomes observed in this study should be interpreted in the context of these population-specific characteristics.

Despite these strengths, several limitations should be considered when interpreting the results. The retrospective design introduces inherent risk of selection bias, and the absence of a control group prevents direct comparison with modular acetabular systems or other bearing combinations. Although the follow-up duration of 6.8 years provides meaningful mid-term data, longer-term observation is necessary to evaluate survivorship, late-onset osteolysis, and the potential for ceramic-related complications over time. Additionally, our results reflect the experience of a single institution, which may limit generalizability. Patient activity level was not systematically recorded in this retrospective cohort and may represent a potential confounding factor when interpreting clinical outcomes and implant performance. Future prospective, multicenter studies incorporating standardized assessments of patient activity level, along with extended follow-up, would be valuable in validating these findings and further clarifying the long-term performance of monoblock ceramic acetabular components.

## 5. Conclusions

The ceramic monoblock acetabular cup demonstrated excellent mid-term clinical and radiologic outcomes with minimal complications in primary THA. Its design allows the safe and consistent use of large-diameter ceramic femoral heads, which may enhance joint stability without compromising fixation. Although the absence of screw holes requires meticulous surgical technique to ensure accurate seating and stable press-fit fixation, our findings suggest that monoblock ceramic cups represent a reliable option for patients undergoing primary THA. Continued long-term follow-up and broader comparative studies will be essential to further define their role in contemporary hip arthroplasty practice.

## Figures and Tables

**Table 1:** Baseline characteristics of the study cohort.

Variable	Value
Sex (Male/Female)	66/40
Age (years)	53.1 (18–85)
Body mass index (kg/m^2^)	24.9 ± 4.0
Follow-up duration (years)	6.8 ± 1.4
Operated Side (Right/Left)	54/52
Surgical Approach	
Minimally invasive surgerytwo-incision	96 (90.6%)
Posterolateral	10 (9.4%)
Diagnosis	
Osteoarthritis	20 (18.9%)
Osteonecrosis of the femoral head	70 (66.0%)
Fracture	8 (7.5%)
Legg-Calve-Perthes disease	1 (0.9%)
Sequelae of developmental dysplasia of the hip	3 (2.8%)
Inflammatory arthritis	3 (2.8%)
Sequelae of septic hip	1 (0.9%)

**Table 2:** Implant size.

Cup Size (mm)	n (%)	Head Size (mm)	n (%)	Stem	n (%)
42	2 (1.9%)	32	8 (7.5%)	ML taper	100 (94.3%)
44	6 (5.7%)			Wagner Cone	4 (3.8%)
46	8 (7.5%)	36	28 (26.4%)	Fitmore	2 (1.9%)
48	20 (18.9%)				
50	21 (19.8%)	40	47 (44.3%)		
52	25 (23.6%)				
54	19 (17.9%)	44	23 (21.7%)		
56	5 (4.7%)				

**Table 3:** Preoperative and last follow-up clincal outcomes & complications.

Variable	Pre-Operative	Last Follow Up	*p*-Value
HHS	37.0 ± 13.4	90.8 ± 6.2	<0.001
WOMAC	66.6 ± 11.5	7.6 ± 6.7	<0.001
VAS	6.45 ± 1.1	1.1 ± 0.8	<0.001
Complication			
Cup fixation failure		1 (0.9%)	
Cup loosening		0	
Squeaking		0	
Ceramic fracture		0	
Stem loosening		1 (0.9%)	
Infection		1 (0.9%)	
Femoral stem periprosthetic fracture		1 (0.9%)	

HHS = Harris Hip Score; WOMAC = Western Ontario and McMaster Universities Osteoarthritis Index; VAS = Visual Analog Scale.

**Table 4:** Radiological Outcome.

Variable	Value/n (%)
Osteolysis	0
Radiolucent line	
Zone 1	2 (1.9%)
Zone 2	2 (1.9%)
Zone 3	0
Acetabular medial surface-cup gap	
Absent (0 mm)	92 (86.8%)
Present (>0 mm)	14 (13.2%)
Gap distance *	3.2 ± 1.4

* Gap distance was calculated only in hips in which a medial acetabular wall–cup gap was present.

**Table 5:** Comparison of Clinical & Radiological Outcomes According to Presence of Medial Gap.

Variable	Gap (n = 14)	No Gap (n = 92)	*p*-Value
Final HHS	90.7 ± 5.7	90.8 ± 6.3	0.950
Final WOMAC	8.6 ± 4.9	7.4 ± 6.9	0.544
Final VAS	0.6 ± 0.6	0.6 ± 0.8	0.881
Radiolucent Line			0.084
Present	2 (14.3%)	2 (2.2%)	
Absent	12 (85.7%)	90 (97.8%)	

HHS = Harris Hip Score; WOMAC = Western Ontario and McMaster Universities Osteoarthritis Index; VAS = Visual Analog Scale.

## Data Availability

The datasets generated and/or analyzed during the current study are not publicly available due to institutional privacy policies and patient confidentiality, but are available from the corresponding author upon reasonable request.
